# An isolated Posterolateral corner injury with rotational instability and hypermobile lateral meniscus: a novel entity

**DOI:** 10.1186/s40634-020-00313-y

**Published:** 2020-12-01

**Authors:** Kazumi Goto, Victoria Duthon, Jacques Menetrey

**Affiliations:** 1 Centre for Sports Medicine and Exercise, Swiss Olympic Medical Center, Hirslanden Clinique La Colline, Chemin Thury 7 A, 1206 Geneve, Switzerland; 2 University Hospital of Geneva, Geneva, Switzerland

**Keywords:** Posterolateral corner, Hypermobile lateral meniscus, Rotational laxity, Popliteomeniscal fascicle

## Abstract

**Purpose:**

Although complete tear of the knee posterolateral corner (PLC) commonly occurs in combination with other knee ligamentous injuries, the incidence of isolated PLC injury was reported only 28% and overlooked in many cases. Nevertheless, an isolated PLC injury does not only provoke posterolateral instability, but also may be associated to hypermobile lateral meniscus. This study aims at showing the characteristics of isolated PLC injuries and to alert potential overlooked cases by describing their arthroscopic findings and clinical characteristics.

**Methods:**

Seventy-one patients with a clinically proven isolated PLC injury who underwent knee arthroscopy were included in this study. Pre-operative symptoms and clinical signs at examination were recorded: Pain at the posterolateral aspect, feelings of instability, catching, locking; and for clinical signs: McMurray test, varus stress test in extension and at 30° of flexion, posterolateral drawer test at 30° and 80°, dial test at 30° and 80° of flexion. In terms of arthroscopic findings, systematic meniscal stability was performed to evaluate the presence of hypermobile lateral meniscus, “lateral drive through test” was also recorded in all cases.

**Results:**

Positive Lateral Drive through test was found in 69 patients (95.8%). Hypermobile lateral meniscus was seen in all patients.

**Conclusions:**

Hyper mobile lateral meniscus was concomitant with all isolated PLC injuries in our case series. As the typical arthroscopic characteristic, lateral drive through test positive were seen in 95.8%. In order to prevent overlooking this concomitant pathology, meticulous arthroscopic observation is crucial.

**Level of evidence:**

Level IV.

## Background

The posterolateral corner (PLC) of the knee is the main restraint to varus forces of the tibia relative to the femur [3]. In spite of this important function, there is still a limited understanding of the structures, biomechanics, and treatment option [4, 7, 25]. Stabilizers of the PLC include the lateral collateral ligament (LCL), the popliteus tendon (PT), the popliteofibular ligament (PFL), and popliteomeniscal fascicles (PMFs) [37]. The PMFs consists of 3 fascicles: anteroinferior, posterosuperior, and posteroinferior [1], which play a role in rotational knee stability and stabilize the lateral meniscus [34, 35]. Isolated posterolateral laxity lesions, as classified Fanelli and Larson classification type A – B (Table 1) [8] or Hughston classification grade I/II (Table 2) [15], have been regarded as rare pathology since PLC injuries are usually associated with anterior cruciate ligament (ACL) or posterior cruciate ligament (PCL) injury, and the incidence of isolated lesion was reported up to 12–28% of all PLC injuries [12, 29]. Therefore, isolated PLC injuries should be overlooked in many cases (50–76%) [23, 24, 29, 37], and this under-recognition may potentially lead to persistent knee posterolateral pain and/or instability sensation [22]. In addition, the post-traumatic disruption of PMFs near the PT may provoke hypermobile lateral meniscus [11, 20, 34]. The hypermobile lateral meniscus may cause pain in the lateral compartment of the knee and mechanical symptoms such as catching, locking and giving way [29]. Apparently, these two pathologies are not independent of each other and may likely exist concomitantly. This study aims at showing the characteristics of isolated PLC injuries defined as type B or grade I/II injury by stating clinical presentation, clinical examination, imaging and arthroscopic findings. The hypothesis of this study was that symptomatic isolated PLC injuries are frequently combined with hypermobile lateral meniscus.

**Table 1:** The Fanelli and Larson classification: classification of damage in posterolateral structures

Classification	Scale of damage	Damaged structure
Type A	10° increase in external rotation of the tibia	PFL, popliteus tendon
Type B	10° increase in external rotation of the tibia	PFL, popliteus tendon
	Slight varus relaxation (5–10 mm increase in varus load test)	LCL
Type C	10° increase in external rotation of the tibia	PFL, popliteus tendon
	Slight varus relaxation (> 10 mm increase in varus load test)	LCL, capsule avalusion, cruciate ligament

*PFL* popliteofibular ligament, *LCL* lateral collateral ligament

**Table 2:** The Hughston classification: classification of posterolateral instability assessed by varus instability

Classification	Varus instability	PCL injury
Grade I	0 – 5 mm	Intact PCL
Grade II	5 – 10 mm	Intact PCL
Grade III	> 10 mm (soft endpoint)	PCL rupture

*PCL* posterior cruciate ligament

## Methods

This study used a retrospective case series design, approved by our institutional review board. Between 2015 and 2019, 204 patients diagnosed as PLC injury underwent surgical treatment in our institution. Of these patients, 71 patients (35 males and 36 females, 72 knees) were matching the inclusion criteria of this study which was to have been diagnosed as isolated type B or grade I/II PLC injury clinically. Exclusion criteria included asymptomatic patients, concomitant knee ligament injury, other meniscal lesion, concomitant chondral lesion, knee dislocation, previous PLC injury, previous history of lateral meniscal lesion, and prior trauma around the knee. All data were collected and analyzed retrospectively.

### Mechanism of injury

We systematically recorded the mechanism of injury when the patient was capable of recollecting it.

### Physical examination

First of all, the physical examination was preceded by careful history taking in every case. Typical symptoms, including history of posterolateral pain, medial or lateral joint line pain, and sensation of instability, were all recorded in our data base. The presence of pain or discomfort by palpation of the joint line was also reported especially around the hiatus popliteus. Grinding and McMurray test [6] were routinely performed. In addition to a comprehensive physical examination of the knee, the following three tests were systematically performed: The varus stress test [21] was performed at both 0° and 30° of knee flexion in supine position. The posterolateral drawer test [2, 15] was performed with the patient supine at 30° and 80° of flexion. The tibia was compressed into posterior with the foot was fixed as externally rotated 15°. The test was repeated at least two times on both 30° and 80° knee flexion. When the amount of increased posterolateral translation was larger than the contralateral side was defined as a positive sign. The dial test [26, 27] was performed in prone position with the knee flexed at 30° and 80°. The tibia was rotated externally to assess the side to side difference. The test was considered positive when there is more than 10° of external rotation in the injured knee compared to the uninjured knee.

### Radiological evaluation

All knees underwent magnetic resonance imaging (MRI) examinations using a knee coil. In fact, most of the patients were coming to our consultation with MRI examinations already performed. These MRI examinations were evaluated by experienced musculoskeletal radiologists and all their reports were reviewed. As one of the patient selection process, those who showed on MRI cruciate ligament injury or abnormal findings of other knee joint structures, such as other meniscal tears or chondral lesions were excluded.

### Arthroscopic evaluation

**Fig. 1:**
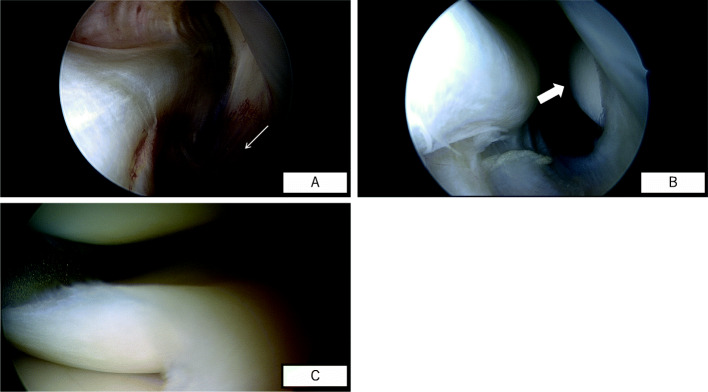
**a**. Drive through test positive on a right knee. **b**. Crescent moon sign. **c**. hypermobile lateral meniscus on a left knee. Arthroscopic findings of the right knee; the image is viewed from the anterolateral portal. **a**: Arthroscopic view, lateral gutter, right knee, of torn popliteomeniscal fascicles (white narrow arrow). **b**: Arthroscopic view, lateral gutter, right knee, showing “crescent moon sign” (white broad narrow). **c**: Arthroscopic view of disruption of the popliteomeniscal fascicles of the lateral meniscus in a left knee. Significant subluxation of the lateral meniscus can be demonstrated by anteromedial traction applied by a surgical probe

Based on the results of the clinical history, the physical examination and radiological evaluation, those corresponding to type B and/or grade I/II (according to diagnostic criteria in Tables 1, and 2) were clinically diagnosed as isolated PLC injury. All those patients have failed previous conservative treatment including all physical therapy modalities. In all patients, arthroscopic evaluation was performed in supine position. The knee was placed at 90° of flexion with a foot support to allow for full range of knee motion. Firstly, a thorough physical examination was performed under anesthesia including varus stress test, posterolateral drawer test, and dial test and compared to contralateral knee in all patients. Then, a standard diagnostic arthroscopy was performed with a 30° arthroscope. The presence of a meniscal tear and its pattern were evaluated by probing the meniscal lesions and recorded. To proceed to the specific evaluation of the lateral meniscus and hiatus popliteus, the arthroscope was introduced through the anterolateral portal into the lateral gutter with the knee in full extension. In this position, the optical lens was rotated to allow for good visualization of the hiatus popliteus and the border of lateral tibial plateau. When the scope was able to be inserted into the hiatus popliteus itself and passed in front of the tendon from anterior to posterior, the “lateral drive-through test” was considered positive [9, 10] (Fig. 1a). Furthermore, when the cartilage border of the lateral tibial plateau was seen as large crescent shape, this sign was called the “crescent moon sign” (Fig. 1b). The presence of these signs was recorded in all patients. After this procedure, the stability of the lateral meniscus was systematically assessed by pulling on the posterior root, posterior horn, posterior and anterior part of the hiatus popliteus. When the lateral meniscus could be subluxated to the middle of the lateral femorotibial compartment (Fig. 1c), it was diagnosed as hypermobile lateral meniscus. A 70° arthroscope was never used in any of these cases.

## Results

A total of 71 patients diagnosed as an isolated PLC injury underwent arthroscopic procedures (35 males and 36 females, 72 knees) in this study. Patient characteristics are shown in detail in Table 3. The mean height was 175.4 ± 9.2 cm (range: 161–191 cm) and the mean weight was 70.2 ± 14.3 kg (range: 45–100 kg). The mean patient age was 32.1 ± 12.8 years (range: 14–73 years). The mean duration between trauma and arthroscopic evaluation was 16.7 ± 23.5 months. Fifty-six patients (77.8%) were to low energy sports related injuries (Ski 14, Football 4, Ice-hockey 4) and twelve (21.4%) were minor knee sprain in daily living or uncertain mechanism, sometimes just tripping. The varus stress test in extension, posterolateral drawer test at 30° of flexion, and the dial test at 30° of flexion were positive in all patients (Table 4).

**Table 3:** Demographic characteristics of the study cohort

Number of cases/knees	71/72
Age, years (range)	32.1 ± 12.8 (14–73)
Sex (male/female)	35/36
Right/Left knee	25/47
The mean duration between arthroscopic diagnosis and trauma, month (range)	16.7 ± 23.5 (1–180)

**Table 4:** The positive rates of physical examinations and arthroscopic findings

Physical examinations	
Varus stress test	72/72 (100%)
Posterolateral drawer test	72/72 (100%)
Dial test	72/72 (100%)
Arthroscopic findings	
Drive through test	69/72 (95.8%)

In terms of arthroscopic findings, the lateral drive-through tests positive was seen in 69 patients (95.8%). Among 72 knees diagnosed as the isolated PLC injury, hypermobile lateral meniscus was found in all knees (Table 4).

## Discussion

The most important finding of this study was that all the patients diagnosed as isolated PLC injury showed a hypermobile LM with posterolateral rotational instability in our case series. Remarkably, during arthroscopy, a positive lateral drive through test was seen in 69/72 knees (95.8%). This specific arthroscopic finding may help to prevent overlooking for isolated PLC injuries with hypermobile lateral meniscus and better detect this entity.

The association between a positive lateral drive through test and PLC structures injury have been investigated by Feng et al. in a cadaveric study. Positive lateral drive through test was present after the section of popliteofibular ligament and distal popliteus or after the section of medial/posteromedial structures (sMCL, deep MCL and POL) or cruciate ligaments (ACL and PCL) [10]. In their study, individual sectioning of any single structure of the PLC could not lead to positive lateral drive through test. In our patients, injury of medial structures or cruciate ligaments has been excluded.

In previous studies, PLC injuries have been classified following two classifications: Fanelli and Larson and Hughston classification (Tables 1, and 2) [8, 15]. The PLC injury we report here might be classified as Fanelli type B injury or Hughston grade I/II injury. Conservative therapy may be a good treatment option despite the lack of solid evidence [32]. In a few studies, good outcomes have been reported after non-operative treatment for grade I and II injuries, however residual lateral laxity was commonly noted in grade II injuries [18, 19]. All patients included in this study have failed with conservative treatment prior to arthroscopy and/or surgical treatment, who were still complaining of posterolateral pain and instability.

Previous reports have showed that injuries of PMFs structures can provoke hypermobile lateral meniscus. Hypermobile lateral meniscus is a relatively uncommon condition and most of the patients typically have no history of associated trauma [11, 14, 28]. Most patients complained of catching, clicking, or sometimes locking in hyperextension as typical symptoms [17], however a few patients complained only of pain without mechanical symptoms [38]. As far as we know, there are no study that investigated this concomitant lesion between grade II PLC injury and hypermobile lateral meniscus.

One of the factors that contributed to the concomitant hypermobile LM in all of our cases was likely a complication of PMF injury. Some anatomical studies showed the strong association between PMFs and hypermobile lateral meniscus. The posterior horn of the lateral meniscus has only a loose attachment to the capsule, which is constructed by the posterior superior popliteomeniscal fascicle and anterior inferior popliteomeniscal fascicle [5]. The superior fascicle arises from the medial fibers of the aponeurosis of the popliteus tendon, whereas the inferior fascicle is a coronary ligament that extends from the meniscus to the edge of the tibia [16]. In particular, the anteroinferior fascicle had a greater degree of control over lateral meniscus [36]. Therefore, even minimal trauma can result in complete failure of these structures and load to subluxation of lateral meniscus in certain subjects [11], which may lead to the pain in the lateral compartment of the knee and mechanical symptoms such as locking and giving way [13, 31].

La Prade et al. [22] reported six patients with isolated tears of the PMFs who had lateral joint line knee pain. All of those patients showed hypermobile lateral meniscus due to tears of the PMFs on arthroscopic examination. In their study, open repair surgeries were performed as complete resolution of their lateral compartment knee pain. Moreover, it was also reported that the “figure-4 test” was positive in all patients as clinical examination, which should be useful to diagnosis isolated PMFs tear. However, it was not clearly mentioned if rotational instability was present in their cohort. Simonian et al. [34] also reported about three cases of lateral meniscus subluxation and they identified a disruption of the fascicular attachments between the popliteus tendon and lateral meniscus as the cause of meniscus instability. In a biomechanical study by Simonian et al. [33], the disruption of the PMFs showed abnormal meniscal motion of approximately doubled compared to intact condition. Therefore, disruption of the PMFs can provoke hypermobile lateral meniscus. Additionally, several therapeutic studies showed that surgical repair restored meniscal stable motion and no recurrences of symptoms were observed [17, 28, 38], which also would support the correlation between symptomatic isolated PLC injury and hypermobile lateral meniscus.

Regarding the contribution of MRI in diagnosing the entity, we and others have to admit that MRI is not very useful except if a specific plan is used to acquire the images [30]. In previous reports, popliteomeniscal fascicle tears were often difficult to recognize and diagnose with MRI examinations [22, 31]. Another study by Simonian et al. reported about 3 patients whose were found to have unstable popliteomeniscal fascicle tears at the time of surgery and had normal MRI findings [34]. Suganuma et al. evaluated popliteomeniscal fascicle in MRI findings of 238 knees including 16 knees with recurrent subluxation of the LM and 215 healthy knees [35]. In their study, abnormal findings of superiorinferior popliteomeniscal fascicles and inferioranterior popliteomeniscal fascicles were noted in 40% and 26% of the control group respectively; and in 100% of the LM hypermobility group. However, the acquisition of their MRI images was performed in the anteromedial-to-posterolateral directed 45° oblique coronal plan, which is not routinely performed in most of institutions. Finally, MRI is a static examination and may just not be appropriate to accurately diagnose LM and posterolateral rotational instability. Therefore, history of the patient and clinical examination are key factors to diagnose this entity.

This study has some limitations. First, the number of patients was small, and the results might vary from those of studies with larger sample size. Secondary, this was a retrospective study which reported characteristic findings of this combined lesion, but this is the first study that reports about the concomitant injury between hypermobile LM and abnormal rotational PLC instability. Finally, there may be asymptomatic hypermobile lateral meniscus or lateral drive through test positive as normal variant in some cases. However, all patient of this study had symptoms and clinical signs as well as abnormal findings during the arthroscopic evaluation. A better understanding of the clinical presentation and the characterization of the arthroscopic examination would help to prevent oversight and provide the proper treatment for isolated PLC injuries with hypermobile LM.

## Conclusion

Isolated PLC injury may comprise lateral meniscal hypermobility with rotational PLC instability and may present pain and instability of the posterolateral compartment of the knee. It is crucial to acknowledge this entity, which patient history, sometimes a minor sport and/or domestic accident, specific clinical examination and meticulous arthroscopic evaluation (including lateral drive through test and palpation of LM) can be key roles.

## Data Availability

The datasets during and/or analyzed during the current study available from the corresponding author on reasonable request.

## Abbreviations

PLC: Posterolateral corner
LM: Lateral meniscus
PMF: Popliteomeniscal fasicle
MRI: Magnetic resonance imaging
AS: Arthroscopy
